# Effectiveness of Interventions to Improve Digital Health Literacy in Forced Migrant Populations: Protocol for a Mixed Methods Systematic Review

**DOI:** 10.2196/50798

**Published:** 2023-11-02

**Authors:** Achille Roghemrazangba Yameogo, Carole Délétroz, Maxime Sasseville, Samira Amil, Sié Mathieu Aymar Romaric Da, Patrick Bodenmann, Marie-Pierre Gagnon

**Affiliations:** 1 Faculté des Sciences Infirmières Université Laval Québec, QC Canada; 2 VITAM - Centre de Recherche en Santé Durable Quebec, QC Canada; 3 School of Health Sciences University of Applied Sciences and Arts Western Switzerland Avenue de Beaumont 21, 1011 Lausanne Switzerland; 4 Department of Vulnerabilities and Social Medicine Unisanté Lausanne Switzerland; 5 Faculty of Biology and Medicine, Vice-Dean Teaching and Diversity University of Lausanne Lausanne Switzerland

**Keywords:** intervention, digital health literacy, forced migrant populations, health literacy, digital literacy, migrant, migrants, immigrant, immigrants, knowledge synthesis, review methods, review methodology, systematic, eHealth literacy

## Abstract

**Background:**

Digital health literacy is considered a health determinant that can influence improved health and well-being, health equity, and the reduction of social health inequalities. Therefore, it serves as an asset for individuals to promote their health. However, low digital health literacy is a major problem among forced migrant populations. They do not always have the capacity and skills to access digital health resources and use them appropriately. To our knowledge, no studies are currently available to examine effective interventions for improving digital health literacy among forced migrant populations.

**Objective:**

This paper presents the protocol for a systematic review that aims to assess the effectiveness of digital health literacy interventions among forced migrant populations. With this review, our objectives are as follows: (1) identify interventions designed to improve digital health literacy among forced migrant populations, including interventions aimed at creating enabling conditions or environments that cater to the needs and expectations of forced migrants limited by low levels of digital health literacy, with the goal of facilitating their access to and use of eHealth resources; (2) define the categories and describe the characteristics of these interventions, which are designed to enhance the abilities of forced migrants or adapt digital health services to meet the needs and expectations of forced migrant populations.

**Methods:**

A mixed methods systematic review will be conducted according to the PRISMA-P (Preferred Reporting Items for Systematic Review and Meta-Analysis Protocols) checklist. The research will be conducted in an iterative process among the different authors. With the help of a medical information specialist, a specific search strategy will be formulated for the 6 most relevant databases (ie, MEDLINE, Embase, CINAHL, Web of Science, Academic Search Premier, PsycINFO, and the Google Scholar search engine). A literature search covering studies published between 2000 and 2022 has already been conducted. Two reviewers then proceeded, individually and independently, to conduct a double selection of titles, abstracts, and then full texts. Data extraction will be conducted by a reviewer and validated by a senior researcher. We will use the narrative synthesis method (ie, structured narrative summaries of key themes) to present a comprehensive picture of effective digital health literacy interventions among forced migrant populations and the success factors of these interventions.

**Results:**

The search strategy and literature search were completed in December 2022. A total of 1232 articles were identified. The first selection was completed in July 2023. The second selection is still in progress. The publication of the systematic review is scheduled for December 2023.

**Conclusions:**

This mixed methods systematic review will provide comprehensive knowledge on effective interventions for digital literacy among forced migrant populations. The evidence generated will further inform stakeholders and aid decision makers in promoting equitable access to and use of digital health resources for forced migrant populations and the general population in host countries.

**International Registered Report Identifier (IRRID):**

DERR1-10.2196/50798

## Introduction

### Overview

Forced migration is a growing global phenomenon. It is defined by the International Organization for Migration [1] as “a non-voluntary, coerced and suffered migratory movement, caused by various factors, but involving the use of force, coercion.” According to Keely and Kraly [2], the reasons for forced migration include wars and armed conflicts, persecution and violence, human rights violations, climate change, natural disasters, and famine. In 2021, there were approximately 1 billion migrants worldwide, accounting for 1 in 7 people in the world [3,4]. Of this population, 82.4 million are forced migrants [3]. An estimated 48 million are internally displaced, 26.4 million are refugees, and 4.1 million are asylum seekers [3]. On host lands or countries, migrants often live with minimal public services and face many complex problems [5]. Due to a lack of regular migration status, stigmatization, xenophobia, racism, discrimination, language and cultural barriers, as well as low income levels, forced migrants have limited access to social and health services and health promotion interventions [6-8]. All these difficulties negatively affect their physical and mental health and well-being and make them more vulnerable [6,9].

The use of information and communication technologies (ICTs) for health, called digital health or eHealth [10,11], could be a promising avenue to address the challenges faced by these forced migrant populations, including internally displaced persons, refugees, asylum seekers, and economic, political, or climate migrants [2,12-17]. Indeed, digital health technologies could play an important role in preventing and promoting the health and well-being of forced migrant populations [18,19]. Chae et al [20] and Wang and Yu [21] explain that forced migrant populations use digital technologies as tools or sources of health information to circumvent barriers in host countries. For example, the internet is regularly used as a cost-effective or free alternative route to search for web-based health information [20]. The study by Chae et al [20] found that about 3%-6% of surveyed women of Korean descent living in the United States used the internet as their primary source for health information. Accessing health information via the internet can also overcome language barriers by using either the language of the country of origin or the language of the host country [21].

Smartphones and digital platforms are digital solutions that could help forced migrant populations understand and connect with complex health systems in host countries [12,17,22]. These digital tools allow these populations, who are unfamiliar with the organization of health systems in host countries, to find doctors, clinics, and hospitals [17]. They also facilitate access to health services, including appointment scheduling and geolocation, especially those in close proximity to their living environment [17]. For example, digital platforms like the Ssyla Digital Therapy Platform, an initiative based in the United Kingdom, connect refugees and migrants to a global network of mental health therapists [12].

In the face of various stressors and challenges experienced by migrants in host countries, digital technologies are resources contributing to improving their well-being. Digital forced migrant resilience was repeatedly linked to social ties [17,23]. Social networks are digital spaces for forced migrant populations to strengthen and create cultural ties (or shared identities) and gather emotional and social support [23]. Connecting with members of their community who are already settled in the host country strengthens feelings of security and trust, belonging to a community, social inclusion, a sense of value, recognition of social status, and nondiscrimination [23]. In addition, contact via mobile phone and social media with family and relatives back home is an important social support for forced migrants, allowing them to overcome physical barriers as well as feelings of social isolation and manage stress [17,24].

Although ICTs have many opportunities and benefits, there are barriers that could prevent forced migrant populations from accessing and using digital health technologies. The first level of difficulties faced by forced migrants is accessing digital media and equipment (eg, internet, computers, tablets, and phones, as well as necessary infrastructure). For example, asylum seekers in Australia were unable to access the internet due to the unaffordable cost or unavailability [25]. Afghan migrants in Iran could not access mobile devices and networks due to infrastructure and legal restrictions [26].

The second category of barriers faced by forced migrants is related to their ability to use available digital health resources. Forced migrants with adverse personal characteristics (eg, advanced age, cognitive impairment, lack of experience, and lack of digital skills) face more barriers to using the internet and apps [27]. For example, older migrants whose health is deteriorating face more barriers to internet use than their healthier counterparts. According to Kouvonen et al [27], the most frequently mentioned challenges were that these digital tools were too complicated or difficult to learn and they presented security challenges.

Finally, the third category of barriers faced by forced migrants is related to their ability to search, find, understand, evaluate, and use health information via the internet. Indeed, language barriers, the complexity of the organization of health systems in host countries, and certain medical terminologies limit access to web-based services for forced migrant populations [28-31]. Moreover, faced with the flood of information on social media, forced migrants do not always have the skills to assess and differentiate between reliable and unreliable health information [32]. This category of barriers is also observed in the general population of the host land or country [33].

Thus, low digital health literacy is an important issue among forced migrant populations [29,34]. Norman and Skinner [35] define digital health literacy as “the ability to search, find, understand and evaluate health information from numerical, electronic sources and use the information to make decisions about one’s health.” In the face of constant digital evolution and the increasing complexity of society and health care systems, forced migrants require additional skills, such as the ability to recognize information needs, trust web-based health information, and interact with the digital health system to improve their health and well-being [36]. To do so, several authors [37-39] indicate that there is a need to conceptualize digital health literacy by considering dimensions such as context (eg, the ability to recognize the existence of an information need and trust web-based health information) and the interactions between migrants and the digital health system. Low levels of digital health literacy could lead to poor health and well-being outcomes [40]. In addition, they may exacerbate disparities (or digital inequalities) in accessing and using digital health services (or digital exclusion), and above all, they may contribute to health inequalities between communities [33]. Low levels of digital health literacy are not conducive to health equity.

To address these major challenges, many initiatives aimed at promoting better health behaviors among forced migrant populations have been developed by different actors [13,37,40]. A comprehensive understanding of these interventions to support digital health literacy among forced migrant populations and their effectiveness is essential to enable policy makers to develop programs and interventions tailored to their needs. However, to our knowledge, there is limited literature on interventions favorable to the digital health literacy of forced migrant populations, which underscores the interest in a research protocol for a mixed methods systematic review.

### Objectives

The overall aim of the systematic review is to assess the effectiveness of interventions aimed at improving digital health literacy among forced migrant populations. To achieve this general objective, 2 specific objectives are pursued, as follows:

- Identify interventions designed to improve digital health literacy among forced migrant populations, including interventions aimed at creating enabling conditions or environments that cater to the needs and expectations of forced migrant populations limited by low levels of digital health literacy, with the goal of facilitating their access to and use of eHealth resources.
- Define the categories and describe the characteristics of these interventions, which are aimed at improving the abilities of forced migrants or adapting digital health services to meet the needs and expectations of forced migrant populations limited by low levels of digital health literacy.

## Methods

### Reporting Standards

This systematic review protocol uses the PRISMA-P (Preferred Reporting Items for Systematic Review and Meta-Analysis Protocol) checklist [41]. The systematic review will be conducted by a research team in accordance with the PRISMA (Preferred Reporting Items for Systematic Reviews and Meta-Analyses) checklist for systematic reviews [42].

### Eligibility Criteria

Eligibility criteria will be based on the PICOS (population, intervention, comparison, outcomes, and study design) model [43] and are described in Table 1. To be selected for review, studies should target forced migrant populations, including internally displaced persons; refugees; asylum seekers; as well as political, economic, and climate migrants. In addition, all studies on interventions related to the theme will be included (ie, if they focus on interventions aimed at digital health literacy among vulnerable migrant populations). As for the types of studies, there will be no restrictions. All quantitative empirical studies, qualitative or mixed methods studies, and studies with or without a control group will be included without distinction. We will consider only studies published in English or French. This systematic review will cover the period from 2000 to 2022. Frank [44] introduced the concept of “digital health” in the early 2000s. We will exclude editorials, commentaries, conference abstracts, protocols, and test recordings.

**Table 1:** Eligibility criteria based on the PICOS (population, intervention, comparison, outcomes, and study design) model [1,2,45].

PICOS model categories	Description
Population (P)	The population consists of forced migrant populations, including internally displaced persons, refugees, asylum seekers, as well as political, economic, and climate migrants. It should be noted that forced migrant populations research is challenged by the diversity of terminology and definitions used [2]. For the purposes of this systematic review, we will rely on definitions from the International Organization for Migration [1] glossary, as follows: Internally displaced persons are “persons forced to flee or leave their homes or places of habitual residence within their own country, including as a result of or to prevent the effects of conflict, violence, human rights violations or natural or man-made disasters” (free translation) [1]. Refugees (convention 1951) means to refer to any “person who, owing to a well-founded fear of being persecuted for reasons of race, religion, nationality, membership of a particular social group or political opinion, is outside the country of his nationality and who is unable or, owing to such fear, does not want to claim the protection of this country” (free translation) [1]. Asylum seeker refers to a “person seeking international protection. In countries with individualized examination procedures, the asylum seeker is a person whose asylum application has not yet been the subject of a final decision by the potential host country. Not every asylum seeker is necessarily recognized as a refugee at the end of the process, but every refugee has initially been an asylum seeker” (free translation) [1]. Migrants include all persons who leave their place of habitual residence to settle, temporarily or permanently, either in another region within the same country or in another country, thus crossing an international border, and for various reasons [1]. When people move in search of a better life or work, we speak of economic migration. In addition, when they migrate to escape persecution due to their political opinions, it is referred to as political migration [1]. Climate migration means to refer to any “movement of a person or group of persons who, essentially for reasons related to a sudden or gradual change in the environment as a result of climate change, are forced to leave their place of habitual residence, or leave it on their own initiative, temporarily or permanently, to go elsewhere in the territory of a state or across an international border” (free translation) [1]. All studies on interventions related to promoting digital health literacy among forced migrant populations will be included.
Intervention (I)	All studies on interventions related to promoting digital health literacy among forced migrant populations will be included. All types of interventions will be considered and classified according to the “Behaviour Change Wheel” model [45]. Indeed, this model makes it possible to systematically characterize interventions aimed at modifying or changing behavior at the individual, organizational, and societal levels.
Comparative (C)	There will be 2 types of comparisons: A specific intervention to promote digital health literacy in forced migrant populations, compared to no specific intervention or usual services. A specific intervention to promote digital health literacy in forced migrant populations, compared to any other intervention, to promote digital health literacy in these populations.
Outcomes (O)	The following elements will be examined: Categories: the following categories will be reviewed: Level of intervention: individual, group, or mixed Mode of design: theory, evidence, or none Targeted behavior: opportunities, motivations, attitudes, and abilities or skills Characteristics of interventions: interventions will be classified according to the following 9 functions of an intervention (ie, types of interventions) based on the model proposed by Michie et al [45]: education, persuasion, incentive, coercion, training, restriction, environmental restructuring, modeling, and empowerment. Results and success factors of interventions: outcomes related to behavior change, self-care behaviors, and resolution of health problems through interventions will be examined along with their success factors. We will also explore outcomes related to access to basic health information and improved quality of life through digital health technologies.
Study design (S)	As for the types of studies, there will be no restrictions. All quantitative empirical studies, qualitative studies, or mixed methods studies, and studies with or without a control group will be included without distinction.

### Review Question

The review question for this systematic review is the following: what are some effective interventions to improve digital health literacy among forced migrant populations, including internally displaced persons, refugees, asylum seekers, as well as economic, political, and climate migrants?

### Search Strategy

The search strategy will be developed by a research team in collaboration with a librarian from Laval University (FB), who specializes in medical information and is experienced in systematic reviews. The research will be carried out in an iterative process between the different authors. First, the systematic literature search will be conducted in the following relevant bibliographic databases: MEDLINE (OVID), Embase, CINAHL, Web of Science, Academic Search Premier, and PsycINFO. We will also search the Google Scholar search engine. The search terms used will be based on a combination of 2 key concepts, which are “Digital Health Literacy” and “forced Migrant Populations.” Research terms for each of these concepts will be developed from the literature and thesauri. Specific details of the strategies will be presented in the form of tables. Then, the search results will be imported to the web-based collaboration tool Covidence (Veritas Health Innovation) [46], a review management software, where duplicates will be removed using the automation function. Missing duplicates will be removed manually.

### Study Selection and Extraction

The selection of studies will be done in Covidence [46]. Individual reviewers from the research team will perform a double selection of titles and abstracts, then the full text of relevant studies. After study selection, data will be extracted by a reviewer and validated by a senior team member, using an extraction grid designed and pilot-evaluated by the research team. Conflicts or discrepancies will be resolved through discussion and consensus within the research team.

### Bias or Quality Assessment

Quality assessment is used to describe the selected articles and to interpret the data in the synthesis [47]. In this review, the quality of studies will be assessed using the Mixed Methods Appraisal Tool (MMAT). This tool will make it possible to simultaneously evaluate the different types of studies selected, whether qualitative, quantitative, or mixed [47].

### Data Synthesis and Analysis

To synthesize the data, we will use narrative synthesis as a method, regardless of the type of study (eg, quantitative, qualitative, or mixed). We will provide a descriptive synthesis of the results of the included studies. A narrative summary of the main results will be produced. Outcomes related to behavior change, self-care behaviors, and health problem–solving (as a result of using the interventions) and the success factors of these interventions will be presented. Additionally, we will present the results related to access to basic health information and improved quality of life through digital health technologies.

### Ethical Considerations

Ethics approval is not required for this systematic review, as it does not require primary data collection. The protocol will be registered with the International Prospective Registry of Systematic Reviews [48]. The results of this systematic review will be disseminated through publication in an academic journal and scientific conferences.

## Results

The search strategy was completed in December 2022. The literature search identified 1232 studies. The first stage of study selection was completed in July 2023. A total of 82 studies were retained for the second selection, which is still in progress. Publication of the paper is scheduled for December 2023.

## Discussion

### The Main Contributions of This Systematic Review

Although literature reviews on health literacy interventions among migrants exist, they have not explicitly included studies on digital health literacy interventions among forced migrants. Fernández-Gutiérrez et al [49] published a systematic review in 2018 that focused solely on health literacy interventions among migrants and included only 9 studies and targeted health care professionals, including nurses. In 2022, a systematic review published by Fox and colleagues [50] on the same topic reviewed the existing literature documenting randomized controlled trials, including 23 articles. To our knowledge, there is no mixed methods literature review on the subject. Our review is the first attempt to examine interventions to improve digital health literacy among forced migrant populations and their effectiveness and challenges.

### Potential Impact and Future Directions

Digital health literacy or eHealth literacy refers to the basic skills required for individuals to take advantage of digital technologies for the benefit of their own health [51]. The importance of these skills is well established for populations with low digital health literacy, such as forced migrants [52]. These skills enable forced migrant populations to promote their own health and well-being. Because of their vulnerability, it is essential to support the development of the skills needed to search, find, understand, and especially, critically evaluate health information among forced migrant populations. Thus, these populations represent important targets for the development of interventions conducive to their digital health literacy as well as the evaluation of the effectiveness of these interventions. This research aims to provide a synthesis of knowledge and generate evidence that will guide effective interventions promoting digital health literacy and the use of eHealth among forced migrant populations.

Determining the level of digital health literacy in forced migrant populations and associated factors contributes to the development of effective and innovative interventions that meet the needs of these populations [28]. Based on the socioecological model [53], 4 factors are identified: individual, interpersonal, community, and societal. The literature shows that there is considerable variability in the methodology of digital health literacy assessments in forced migrant populations [28]. Chesser et al [28] indicate that some of these measurement tools used for evaluation have inherent limitations. The wide variability in intervention designs to support digital health literacy in forced migrant populations could be explained by the variety of measurement tools and the plurality of associated factors. They also explain why some interventions are more effective than others. There is, therefore, a need to comprehensively understand and highlight effective interventions that support digital health literacy among forced migrant populations and the factors that these interventions have addressed. Mixed methods will be used in this systematic review to provide a broader and more comprehensive picture of the existing literature on effective interventions for improving digital health literacy in forced migrant populations.

### Conclusions

The results of this systematic review will provide a comprehensive picture of effective interventions that promote digital health literacy among forced migrant populations. They will share knowledge and evidence on digital health and the use of eHealth among the forced migrant populations with different stakeholders. This evidence and knowledge, in turn, will aid decision makers in promoting equitable access to and use of digital health resources among forced migrants and the general population in host countries.

## Appendix

Multimedia Appendix 1 Search strategies for each database.

## Abbreviations

ICT: information and communication technology
MMAT: Mixed Methods Appraisal Tool
PICOS: population, intervention, comparison, outcomes, study design
PRISMA: Preferred Reporting Items for Systematic Reviews and Meta-Analyses
PRISMA-P: Preferred Reporting Items for Systematic Review and Meta-Analysis Protocol

## Data Availability

Data relating to the search strategy for this review are available in Multimedia Appendix 1. All data generated or analyzed during this study (including the PRISMA [Preferred Reporting Items for Systematic Reviews and Meta-Analyses] flow chart) will be included as multimedia appendices. Databases created during the data extraction process will be available upon reasonable request.
