# Supplementary material for: Effectiveness of Interventions to Improve Digital Health Literacy in Forced Migrant Populations: Protocol for a Mixed Methods Systematic Review
Source: JMIR Res Protoc. 2023 Nov 2;12:e50798. doi: 10.2196/50798 (PMC10654904; doi:10.2196/50798)
Supplement: Multimedia Appendix 1 [file resprot_v12i1e50798_app1.docx]

**Supplementary materials – Database search strategy**

**Medline (OVID)**

**Date of the search:** 14-12-2022

**Database limit:** no database limit has been applied

| **#** | **Search strategy** | **Results** |
| --- | --- | --- |
| 1 | "eHealth literacy".ti,ab,kw,kf OR "electronic health literacy".ti,ab,kw,kf OR "digital literacy".ti,ab,kw,kf | 1071 |
| 2 | Consumer Health Information/ OR exp Health Literacy/ | 12821 |
| 3 | skill?.ti,ab,kw,kf OR "health* literacy".ti,ab,kw,kf | 233942 |
| 4 | 2 OR 3 | 240124 |
| 5 | Internet/ OR Internet-Based Intervention/ OR Smartphone/ OR Telemedicine/ OR Digital Technology/ OR Mobile Applications/ | 126928 |
| 6 | Telemedicine.ti,ab,kw,kf OR "Mobile Health".ti,ab,kw,kf OR mHealth.ti,ab,kw,kf OR ehealth.ti,ab,kw,kf OR "e-health".ti,ab,kw,kf OR "m-health".ti,ab,kw,kf OR Telehealth.ti,ab,kw,kf OR eHealth.ti,ab,kw,kf  OR Smartphone?.ti,ab,kw,kf OR Internet.ti,ab,kw,kf OR Web.ti,ab,kw,kf OR (information adj2 (retriev* OR Seek* OR search* OR find* OR understand* OR evaluat*)).ti,ab,kw,kf OR ((Health OR eHealth OR mobile) adj4 (application? OR app?)).ti,ab,kw,kf OR digital.ti,ab,kw,kf OR "health information".ti,ab,kw,kf | 498786 |
| 7 | 5 OR 6 | 541782 |
| 8 | Refugees/ OR Transients and Migrants/ OR exp Human Migration/ OR "Emigrants and Immigrants"/ | 51332 |
| 9 | Refuge*.ti,ab,kw,kf OR Asylum.ti,ab,kw,kf OR ((Forced OR involuntary) adj2 migrant*).ti,ab,kw,kf OR Immigra*.ti,ab,kw,kf OR displaced.ti,ab,kw,kf | 95374 |
| 10 | 8 OR 9 | 124850 |
| 11 | (4 AND 7) OR 1 | 21634 |
| 12 | 10 AND 11 | 237 |

**Embase (Embase.com)**

**Date of the search:** 14-12-2022

**Database limit:** results have been limited to prepublications & Embase database only

| **#** | **Search strategy** | **Results** |
| --- | --- | --- |
| 1 | 'ehealth literacy'/de | 269 |
| 2 | "eHealth literacy":ti,ab,kw OR "electronic health literacy":ti,ab,kw OR "digital literacy":ti,ab,kw | 1,068 |
| 3 | #1 OR #2 | 1,199 |
| 4 | 'health literacy'/de OR 'medical information'/de OR 'consumer health information'/de OR 'information seeking'/de OR 'skill'/de | 209,742 |
| 5 | skill$:ti,ab,kw OR "health* literacy":ti,ab,kw | 309,099 |
| 6 | #4 OR #5 | 415,546 |
| 7 | 'Internet'/de OR 'web-based intervention'/de OR 'mobile phone'/exp OR 'telemedicine'/de OR 'telehealth'/de OR 'digital technology'/de OR 'mobile application'/exp | 222,111 |
| 8 | Telemedicine:ti,ab,kw OR "Mobile Health":ti,ab,kw OR mHealth:ti,ab,kw OR ehealth:ti,ab,kw OR "e-health":ti,ab,kw OR "m-health":ti,ab,kw OR Telehealth:ti,ab,kw OR eHealth:ti,ab,kw OR Smartphone$:ti,ab,kw OR Internet:ti,ab,kw OR Web:ti,ab,kw OR (information NEAR/2 (retriev* OR Seek* OR search* OR find* OR understand* OR evaluat*)):ti,ab,kw OR ((Health OR eHealth OR mobile) NEAR/4 (application$ OR app$)):ti,ab,kw OR digital:ti,ab,kw OR "health information":ti,ab,kw | 629,951 |
| 9 | #7 OR #8 | 705,731 |
| 10 | 'migrant'/exp OR 'forced migrant'/exp OR 'refugee'/exp OR 'migration'/exp | 90,830 |
| 11 | Refuge*:ti,ab,kw OR Asylum:ti,ab,kw OR ((Forced OR involuntary) NEAR/2 migrant*):ti,ab,kw OR Immigra*:ti,ab,kw OR displaced:ti,ab,kw | 107,147 |
| 12 | #10 OR #11 | 154,652 |
| 13 | (#6 AND #9) OR #3 | 49,706 |
| 14 | #12 AND #13 | 559 |
| 15 | #14 AND [embase]/lim NOT ([embase]/lim AND [medline]/lim) | 190 |

**CINAHL**

**Date of the search:** 14-12-2022

**Database limit:** no database limit has been applied

| **#** | **Search strategy** | **Results** |
| --- | --- | --- |
| 1 | TI "eHealth literacy" OR AB "eHealth literacy" OR TI "electronic health literacy" OR AB "electronic health literacy" OR TI "digital literacy" OR AB "digital literacy" | 671 |
| 2 | MH "Consumer Health Information+" OR MH "Information Retrieval+" | 30,357 |
| 3 | TI skill# OR AB skill# OR TI "health* literacy" OR AB "health* literacy" | 143,671 |
| 4 | S2 OR S3 | 169,005 |
| 5 | MH Internet OR MH "Internet-Based Intervention" OR MH Smartphone OR MH "Mobile Applications" OR MH Telemedicine OR MH Telehealth OR MH "Digital Technology" | 93,911 |
| 6 | TI Telemedicine OR AB Telemedicine OR TI "Mobile Health" OR AB "Mobile Health" OR TI mHealth OR AB mHealth OR TI ehealth OR AB ehealth OR TI "e-health" OR AB "e-health" OR TI "m-health" OR AB "m-health" OR TI Telehealth OR TI eHealth OR AB Telehealth OR AB eHealth OR TI Smartphone# OR AB Smartphone# OR TI Internet OR AB Internet OR TI Web OR AB Web OR TI (information N2 (retriev* OR Seek* OR search* OR find* OR understand* OR evaluat*)) OR AB (information N2 (retriev* OR Seek* OR search* OR find* OR understand* OR evaluat*)) OR TI ((Health OR eHealth OR mobile) N4 (application# OR app#)) OR AB ((Health OR eHealth OR mobile) N4 (application# OR app#)) OR TI digital OR AB digital OR TI "health information" OR AB "health information" | 207,589 |
| 7 | S5 OR S6 | 254,924 |
| 8 | MH Refugees OR MH "Transients and Migrants" OR MH "Emigration and Immigration" OR MH Immigrants | 35,170 |
| 9 | TI Refuge* OR AB Refuge* OR TI Asylum OR AB Asylum OR TI ((Forced OR involuntary) N2 migrant*) OR AB ((Forced OR involuntary) N2 migrant*) OR TI Immigra* OR AB Immigra* OR TI displaced OR AB displaced | 33,395 |
| 10 | S8 OR S9 | 49,498 |
| 11 | (S4 AND S7) OR S1 | 20,282 |
| 12 | S10 AND S11 | 208 |

**Academic Search Premier**

**Date of the search:** 14-12-2022

**Database limit:** no database limit has been applied

| **#** | **Search strategy** | **Results** |
| --- | --- | --- |
| 1 | DE "DIGITAL literacy" | 1,002 |
| 2 | TI "eHealth literacy" OR AB "eHealth literacy" OR KW "eHealth literacy" OR TI "electronic health literacy" OR AB "electronic health literacy" OR KW "electronic health literacy" OR TI "digital literacy" OR AB "digital literacy" OR KW "digital literacy" | 2,358 |
| 3 | S1 OR S2 | 2,595 |
| 4 | DE "HEALTH literacy" OR DE "ELECTRONIC information resource literacy" OR DE "INFORMATION retrieval" | 38,784 |
| 5 | TI skill# OR AB skill# OR KW skill# OR TI "health* literacy" OR AB "health* literacy" OR KW "health* literacy" | 334,168 |
| 6 | S4 OR S5 | 365,775 |
| 7 | DE "INTERNET" OR DE "MOBILE apps" OR DE "IPHONE mobile apps" OR DE "TABLET mobile apps" OR DE "SMARTPHONES" OR DE "TABLET computers" OR DE "DIGITAL technology" OR DE "TELEMEDICINE" OR DE "MOBILE health" | 152,511 |
| 8 | TI Telemedicine OR AB Telemedicine OR KW Telemedicine OR TI "Mobile Health" OR AB "Mobile Health" OR KW "Mobile Health" OR TI mHealth OR AB mHealth OR KW mHealth OR TI ehealth OR AB mHealth OR KW mHealth OR TI "e-health" OR AB "e-health" OR KW "e-health" OR TI "m-health" OR AB "m-health" OR KW "m-health" OR TI Telehealth OR TI eHealth OR AB Telehealth OR AB eHealth OR KW Telehealth OR KW eHealth OR TI Smartphone# OR AB Smartphone# OR KW Smartphone# OR TI Internet OR AB Internet OR KW Internet OR TI Web OR AB Web OR KW Web OR TI (information N2 (retriev* OR Seek* OR search* OR find* OR understand* OR evaluat*)) OR AB (information N2 (retriev* OR Seek* OR search* OR find* OR understand* OR evaluat*))  OR KW (information N2 (retriev* OR Seek* OR search* OR find* OR understand* OR evaluat*)) OR TI ((Health OR eHealth OR mobile) N4 (application# OR app#)) OR AB ((Health OR eHealth OR mobile) N4 (application# OR app#)) OR KW ((Health OR eHealth OR mobile) N4 (application# OR app#)) OR TI digital OR AB digital OR KW digital OR TI "health information" OR AB "health information" OR KW "health information" | 1,076,333 |
| 9 | S7 OR S8 | 1,103,896 |
| 10 | DE "REFUGEES" OR DE "FORCED migration" OR DE "IMMIGRANTS" OR DE "INTERNAL migration" OR DE "POLITICAL asylum" | 69,812 |
| 11 | TI Refuge* OR AB Refuge* OR KW Refuge* OR TI Asylum OR AB Asylum OR KW Asylum OR TI ((Forced OR involuntary) N2 migrant*) OR AB ((Forced OR involuntary) N2 migrant*) OR KW ((Forced OR involuntary) N2 migrant*) OR TI Immigra* OR AB Immigra* OR TI displaced OR AB displaced OR KW displaced | 217,774 |
| 12 | S10 OR S11 | 238,374 |
| 13 | (S6 AND S9) OR S3 | 37,201 |
| 14 | S12 AND S13 | 313 |

**Web of Science**

**Date of the search:** 14-12-2022

**Database limit:** no database limit has been applied

| **#** | **Search strategy** | **Results** |
| --- | --- | --- |
| 1 | TS="eHealth literacy" OR TS="electronic health literacy" OR TS="digital literacy" | 4,600 |
| 2 | TS=skill$ OR TS="health* literacy" | 519,765 |
| 3 | TS=Telemedicine OR TS="Mobile Health" OR TS=mHealth:ti,ab,kw OR TS=ehealth OR TS="e-health" OR TS="m-health" OR TS=Telehealth OR TS=eHealth OR TS=Smartphone$ OR TS=Internet OR TS=Web OR TS=(information NEAR/2 (retriev* OR Seek* OR search* OR find* OR understand* OR evaluat*)) OR TS=((Health OR eHealth OR mobile) NEAR/4 (application$ OR app$)) OR TS=digital OR TS="health information" | 1,817,370 |
| 4 | TS=Refuge* OR TS=Asylum OR TS=((Forced OR involuntary) NEAR/2 migrant*) OR TS=Immigra* OR TS=displaced | 276,158 |
| 5 | (#2 AND #3) OR #1 | 51,350 |
| 6 | #4 AND #5 | 527 |

**PsycInfo (OVID)**

**Date of the search:** 14-12-2022

**Database limit:** no database limit has been applied

| **#** | **Search strategy** | **Results** |
| --- | --- | --- |
| 1 | Digital Literacy/ | 1018 |
| 2 | "eHealth literacy".ti,ab,id OR "electronic health literacy".ti,ab,id OR "digital literacy".ti,ab,id | 1037 |
| 3 | 1 OR 2 | 1789 |
| 4 | Health Literacy/ | 3878 |
| 5 | skill?.ti,ab,id OR "health* literacy".ti,ab,id | 250412 |
| 6 | 4 OR 5 | 251062 |
| 7 | Internet/ OR smartphones/ OR mobile applications/ OR Telemedicine/ OR Digital Technology/ OR Mobile Health/ | 43707 |
| 8 | Telemedicine.ti,ab,id OR "Mobile Health".ti,ab,id OR mHealth.ti,ab,id OR ehealth.ti,ab,id OR "e-health".ti,ab,id OR "m-health".ti,ab,id OR Telehealth.ti,ab,id OR eHealth.ti,ab,id OR Smartphone?.ti,ab,id OR Internet.ti,ab,id OR Web.ti,ab,id OR (information adj2 (retriev* OR Seek* OR search* OR find* OR understand* OR evaluat*)).ti,ab,id OR ((Health OR eHealth OR mobile) adj4 (application? OR app?)).ti,ab,id OR digital.ti,ab,id OR "health information".ti,ab,id | 145259 |
| 9 | 7 OR 8 | 153636 |
| 10 | human migration/ OR refugees/ OR immigration/ OR Asylum Seeking/ | 39535 |
| 11 | Refuge*.ti,ab,id OR Asylum.ti,ab,id OR ((Forced OR involuntary) adj2 migrant*).ti,ab,id OR Immigra*.ti,ab,id OR displaced.ti,ab,id | 53362 |
| 12 | 10 OR 11 | 61659 |
| 13 | (6 AND 9) OR 3 | 13313 |
| 14 | 12 AND 13 | 190 |

**Google Scholar (**[**https://harzing.com/resources/publish-or-perish**](https://harzing.com/resources/publish-or-perish)**)**

**Date of the last search:** 14-12-2022

**Database limit:** only up to the first 20 results per string have been considered; citations records & patents options have been removed from the search.

| **#** | **Search** | **# Results** |  |
| --- | --- | --- | --- |
| 1 | "eHealth literacy" AND migrant\|migrants\|migration | 20 |  |
| 2 | "electronic health literacy" AND migrant\|migrants\|migration | 20 |  |
| 3 | "digital literacy" AND migrant\|migrants\|migration | 20 |  |
| 4 | "eHealth literacy" AND immigration\|Asylum | 20 |  |
| 5 | "electronic health literacy" AND immigration\|Asylum | 20 |  |
| 6 | "digital literacy" AND immigration\|Asylum | 20 |  |
| 7 | "eHealth literacy" AND refugees\|refuge | 20 |  |
| 8 | "electronic health literacy" AND refugees\|refuge | 20 |  |
| 9 | "digital literacy" AND refugees\|refuge | 20 |  |
|  | **Total number of results** | **180** |  |
